# Eucain

**Published:** 1897-02

**Authors:** J. C. Clemesha

**Affiliations:** Buffalo, N. Y.


ARTICLE V.

### EUCAIN.

BY J. C. CLEMESHA, M. D., M. R. C. S., ENG., L. R. C. P., LOND., BUFFALO, N. Y.

In a paper before the Hufeland Society, Berlin, April 16, 1896, Dr. Vinci, of Messina, described the chemical composition and properties of a new compound, which, for convenience sake, has been called eucain. This new drug, it is stated, is not derived from the vegetable kingdom, but is a laboratory product, and its proper name is methyl-benzoyl-tetramethyl-gamina-oxypiperidine-carbonic acid-methyal-ester, while the salt in use is the muriate which crystallizes from a watery solution in permanent shiny scales and is soluble in water to the extent of 6 per cent. Furthermore it can be prepared in any quantity, and I believe the intention is to put it upon the market at a cost considerably below that of cocain. Dr. Vinci described eucain as possessing the properties of cocain as a local anesthetic, but as being less toxic and as having no effect upon the pupil.

Having read an extract of the above paper, and realizing, if the statements were accurate, that an important addition had been made to the list of drugs used in ophthalmic practice, Dr. Howe procured a supply from the New York agents and a 5 per cent watery solution was prepared. The first day it was used at the Buffalo Eye and Ear Infirmary was in a 5 per cent solution and in the following cases: (1) Removal of a foreign body imbedded in the corner; (2) removal of granulation after a strabismus operation; (3) the operation of tatooing the cornea. In the foregoing cases the patients complained that the instillation was followed by a smarting, pricking sensation, lasting some minutes, while it was noted that some hyperemia of the conjunctiva was produced, which remained for half an hour or so after the anesthesia passed away. Anesthesia was complete in from five to ten minutes, none of the patients complaining of the least pain during operative manipulation. It was also noted that the pupil did not dilate, but reacted well to light, and that the corneal epithelium showed no tendency to become sodden, as it does after the application of cocain.

The day following Dr. Howe used it with complete success in a strabismus operation, and it has been used for extractions and iridectomies, the patients complaining of no pain whatsoever.

Later a 1 to 2 per cent solution was prepared, and it was found that anesthesia was as readily produced without the pricking sensation which follows the application of the stronger solutions, and these weaker solutions are being employed at the infirmary for the removal of foreign bodies in the eye and other minor operations.

Comparison was made with a 2 per cent solution of cocain and a solution of eucain of the same strength, with the result that the two drugs were of the same value as regards rapidity, duration and intensity of the anesthesia.

Eucain has been proved to be less poisonous than cocain. Animals treated with eucain survived, while animals injected with cocain perished. The heart is in no way influenced by it, while it is a well-known fact that dangerous symptoms often arise from the use of cocain. Professor Charteris, of Glasgow, has made comparative experiments with the hydrochlorat of eucain and the hydrochlorat of cocain. Watery solutions of these salts were injected into guinea pigs, beginning with a small dose was accurately ascertained. It was found, after many experiments, that the toxic dose of eucain per kilo body weight was 0.09 gram, and that the toxic dose of cocain per kilo body weight was 0.068 gram. It was also noticed that the physiological action produced by a given dose of eucain did not follow nearly so rapidly as that which followed a similar dose of cocain, hence it was concluded that the action of eucain was slower in onset and less in intensity. Finally, eucain solutions are stable and permanent and do not decompose, like those of cocain when kept for a time.

Tropacocain, another local anesthetic, seems to be midway between cocain and eucain. It is less toxic than cocain, and on stillation produces slight smarting and some hyperemia of the conjunctiva. Mydriasis is usually absent and the anesthesia is more quickly complete and does not last as long as cocain. Unfortunately its manufacture has been abandoned on account of the difficulty of its preparation.

Eucain has also been employed in dentistry for the extraction of teeth; 3 to 5 minims of a 10 per cent solution, injected into each root to be extracted, is sufficient. It has also been used in laryngology and, in the form of an ointment, in painful skin affections.—*Buffalo Medical Journal.*

